# The diagnosis and prevalence of hypoprolactinemia in patients with panhypopituitarism and the effects on depression and sexual functions

**DOI:** 10.1007/s11102-024-01393-0

**Published:** 2024-05-03

**Authors:** Ilknur Uzun, Zuleyha Karaca, Aysa Hacioğlu, Kursad Unluhizarci, Fahrettin Kelestimur

**Affiliations:** 1 Faculty of Medicine, Department of Endocrinology, Erciyes University, Kayseri, Turkey; 2 Faculty of Medicine, Department of Endocrinology, Yeditepe University, İstanbul, Turkey

**Keywords:** Depression, Hypopituitarism, Hypoprolactinemia, Prolactin, Sexual functions, TRH stimulation test

## Abstract

**Purpose:**

We aimed to investigate the prevalence and the diagnostic criteria of hypoprolactinemia in patients with panhypopituitarism and the effects of hypoprolactinemia on depression and sexual functions.

**Materials and methods:**

Forty-eight patients with panhypopituitarism and 20 healthy volunteers were included. Basal hormone levels were measured and a TRH stimulation test was performed. For the evaluation of sexual functions, questionnaries of Female Sexual Functional Index (FSFI) for females and International Erectile Functional Index for males were performed to the subjects. Depressive symptoms were evaluated by Beck Depression Envontory score (BDI-II).

**Results:**

The peak PRL response to TRH stimulation test at 5th percentile in the control group was 18.6 ng/ml in males and 41.6 ng/ml in females and accepted as the cut-offs for sufficient response of PRL. Prolactin was insufficient in 42(87.5%) patients. A basal PRL level of ≤ 5.7 ng/ml in males and 7.11 ng/ml in females was 100% specific in predicting an inadequate response to TRH stimulation test with 80% and 70% sensitivity respectively. A basal PRL level of ≥ 8.5 ng/dl in males was 100% specific and 76% sensitive, and in females a level of ≥ 15.2 ng/dl was 96% specific and 66% sensitive in predicting an adequate response to TRH. PRL deficient patients with panhypopituitarism had higher depression scores compared to the controls, lower sexual function scores in males.

**Conclusion:**

PRL deficiency is prevalent among individuals with panhypopituitarism, with the potential to result in elevated depression scores in both sexes and impaired sexual functions in males. A basal PRL level seems to be sufficient for the diagnosis of hypoprolactinemia in routine clinical practice.

**Supplementary Information:**

The online version contains supplementary material available at 10.1007/s11102-024-01393-0.

## Introduction

Prolactin (PRL) is a polypeptide hormone secreted mainly from the pituitary gland. The pituitary prolactin (PRL) secretion is regulated by hypothalamus in an inhibitory way by dopamine which plays some roles not only in lactation but also other systems [1, 2]. The patients with pituitary disorders may present with either hyper- or hypoprolactinemia or normal prolactin levels. Hyperprolactinemia has been investigated in detail in all aspects in the literature. However, hypoprolactinemia is usually neglected since the diagnosis does not change the clinical management of these patients. In other words, since clinicians do not feel a need to diagnose hypoprolactinemia, the diagnostic criteria are not clearly defined for it. Very recently, we tried to call attention to the importance of hypoprolactinemia in hypopituitarism [3].

Hypoprolactinemia is typically recognized in patients with Sheehan syndrome as the failure of postpartum lactation [4]. However, there are many other physiological actions of PRL including reproduction, immunoregulation, homeostasis, neuroprotection, behavior, water and electrolyte balance, and embryonic and fetal development [1]. Whatever the underlying cause of hypoprolactinemia is, low PRL levels were associated with decreased well-being and sexual functioning [5, 6]. However, almost all present data regarding the effects of PRL deficiency are derived from studies including subjects with dopamine agonist (DA) induced hypoprolactinemia. Unlike DA induced hypoprolactinemia, hypoprolactinemia due to failure of pituitary lactotrophs is suspected to affect dopaminergic tone negatively.

Due to limited data availability, we decided to investigate the prevalence and the diagnostic criteria of hypoprolactinemia in patients with panhypopituitarism and the effects of hypoprolactinemia on depression and sexual functions in the present study.

## Materials and methods

The study was approved by Local Ethics Committee and informed consent was signed by the volunteers. Forty-eight patients who were followed up with a diagnosis of panhypopituitarism in the Endocrinology Clinic and 20 healthy volunteers were included in the study. Patients who were pregnant, lactating, using medications that could affect PRL levels, patients with prolactinoma, acromegaly, active Cushing syndrome, active malignancy, chronic liver or renal insufficiency were excluded from the study.

All patients were under appropriate hormone replacement therapies for hypopituitarism at the time of evaluation. Basal hormone levels were measured in the fasting state in the morning and a TRH stimulation test was performed. PRL levels were measured at baseline and 20 and 60 min after the i.v. injection of 200 µg TRH ferring ampul (GmbH, Kiel, Germany). The diagnosis of hypoprolactinemia was determined by a TRH stimulation test. The peak PRL response to the TRH stimulation test at 5th percentile in the healthy control group, for males and females separately, was accepted as the cut-off for sufficient response of PRL to TRH.

For the evaluation of sexual functions, questionnaries of Female Sexual Functional Index (FSFI) [7] for females and International Erectile Functional Index for males were performed to the subjects [8]. Male sexual functions were categorized as normal for scores 22–25, mild deterioration for 17–21, mild to moderate deterioration for 12–16, moderate deterioration for 8–11, severe deterioration for 5–7 [8]. Depressive symptoms were evaluated by Beck Depression Envontory score (BDI-II). Depressive symptoms were categorized as normal for scores 0–9 mild for 10–18, moderate for 19–29, severe for 30–63 [9].

Body mass index (BMI) was calculated according to formula (body weight (kg)/ height (m)^2^). Glucose, cholesterol and basal hormone levels were measured in the morning after an overnight fast. Serum glucose, triglyceride, total and HDL-cholesterol levels were measured by spectrophotometric enzymatic method (Cobas; Roche Diagnostics, Mannheim, Germany). LDL-cholesterol levels were calculated with the Friedewald formula. TSH, free T3, free T4, FSH, LH, estradiol, testosterone, PRL, cortisol, IGF-1 levels were measured using the electrochemiluminescence immunoassay (ECLIA) technique with commercially available assays (Cobas; Roche Diagnostics, Mannheim, Germany). Serum PRL levels were measured by the ECLIA method based on sandwich complex formation using two monoclonal antibodies specifically against human PRL. The limit of detection, corresponds to the lowest analyte concentration which can be detected, is 0.094 ng/mL. Intra and inter-assay coefficients of variability (CV) are 3.1% and 3.8%, respectively for 3.5 ng/mL PRL level. Intra and inter-assay coefficients of variability (CV) are 1.8% and 2.7%, respectively for 28 ng/mL PRL level.

### Statistical analysis

IBM SPSS Statistics version 22.0 (IBM Inc. Chicago, IL, ABD) was used for analysis. Descriptive data were presented as median (min-max), mean ± standard deviation (SD) according to the distribution of data and frequency. Normal distribution of data was tested by Shapiro-Wilk test. Mann-Whitney U test or Kruskal-Wallis test for comparing continous variables and Chi-square test or Fischer exact test was used for comparing categorical variables based upon the distribution of data. Statistical significancy was set for *p* < 0.05. Area under curve (AUC) of PRL response to TRH was calculated according to trapezoid formula. Correlation analysis was tested with Spearman or Pearson correlation analysis tests according to the distribution of data. To determine a cut-off for basal PRL receiver operating characteristic (ROC)-curve analysis was performed.

## Results

**Fig. 1:**
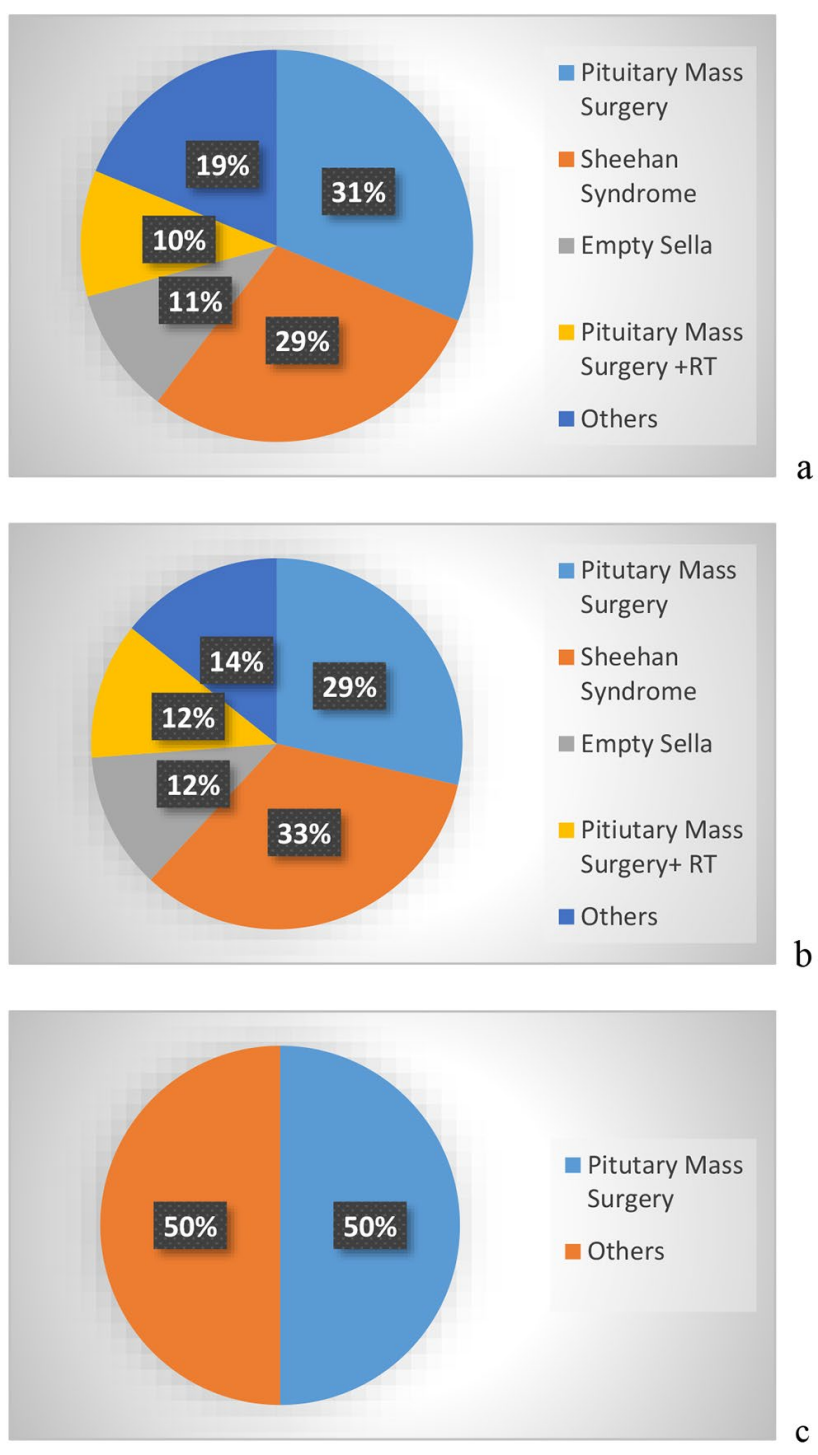
Etiology of panhypopituitarism. (**1a**) in the whole group. Pitutary surgery only (n: 15): Non-functioning pituitary adenoma (n:12) + Craniopharyngioma (n:1) + Meningioma (n:1) + Cushing disease (n:1) Pituitary surgery + RT (radiotherapy (n: 5): Non-functioning pituitary adenoma (n:2) + Craniopharyngioma (n:2) +  Sheehan syndrome (n:14) Empty Sella (n:5) Others (n: 9): Genetic diseases diagnosed in childhood (n:7) + Traumatic brain injury (n:1) + Meningitis (n:1). (**1b**) in patients with hypoprolactinemia. Pitutary surgery only (n:12) Non-functioning pituitary adenoma (n:10) + Craniopharyngioma (n:1) + Cushing’s disease (n:1) Pituitary surgery + radiotherapy (n:5): Non-functioning pituitary adenoma (n:2) + Craniopharyngioma (n:2) + Sheehan syndrome (n:14) Empty sella (n:5) Others (n:6): Genetic diseases diagnosed in childhood (n:5) + Traumatic brain injury (n:1). (**1c**) in patients without hypoprolactinemia. Pitutary surgery only (n: 3): Non-functioning pituitary adenoma (n:2) + Meningioma (n:1). Others (3): Genetic diseases diagnosed in childhood (n:2) + meningitis (n:1)

The causes of hypopituitarism was surgery for a mass lesion in the sellar area in 20 patients (5 of them received consequent radiotherapy), Sheehan syndrome in 14 patients, empty sella in 5, and other causes in 9 patients (Fig. 1). The most common cause of panhypopituitarism was Sheehan syndrome in PRL deficient patients and surgery in PRL sufficient patients. The etiologic distribution of hypopituitarism was found to be similar in PRL deficient and sufficient subjects. All patients had TSH, ACTH, GH and gonadotropin deficiency. All patients were replaced for glucocorticoids and thyroid hormone. All male patients were on testosterone replacement, 4 of 5 female patients who were < 45 years of age were on estrogen replacement, 8 patients were on GH replacement therapy. 5 patients had AVP deficiency and they were on desmopressin replacement therapy.

The peak PRL response to TRH stimulation test at 5th percentile in the healthy control group was 18.6 ng/ml in males and 41.6 ng/ml for females and were accepted as the cut-offs for the sufficiency of PRL. In the patient group, PRL was sufficient in 6 (12.5%) patients and insufficient in 42 (87.5%) patients.

All patients with AVP deficiency had insufficient PRL response to TRH stimulation test. The etiologies of hypopituitarism in all these five patients were surgery followed by radiotherapy (2 for non-functioning pituitary adenoma, 2 for craniopharyngioma, and 1 for Cushing disease).

**Fig. 2:**
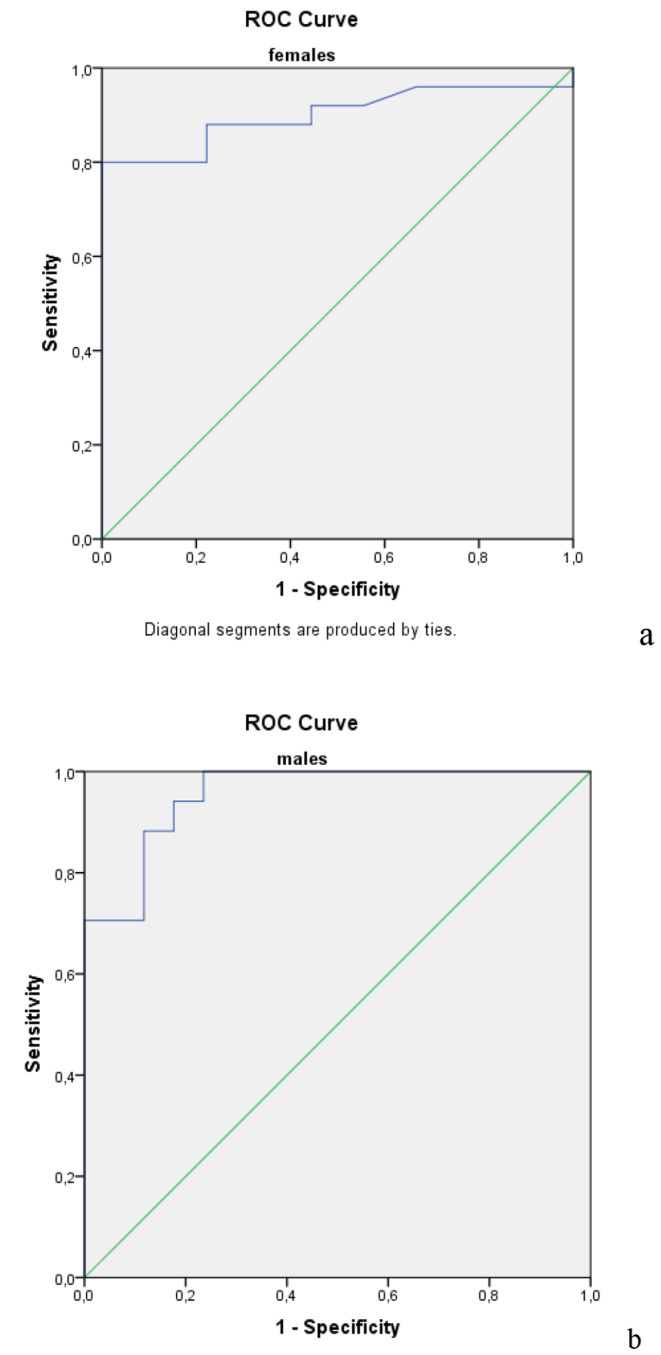
ROC-curves of the peak PRL responses to TRH test in females and males

We performed a ROC curve analysis to define a cut-off level for basal PRL according to the sufficiency of TRH stimulation test (Fig. 2). In males, a basal PRL level of ≤ 5.7 ng/ml was 100% specific, 70% sensitive in predicting an inadequate response to the TRH stimulation test and a basal PRL level of ≥ 8.5 could predict an adequate PRL response to TRH with 100% specifity and 76% sensitivity. In females, a basal PRL level of ≤ 7.11 ng/ml was 100% specific, 80% sensitive in predicting an inadequate response to the TRH stimulation test and a cut-off level ≥ 15.2 ng/dl was 96% specific and 66% sensitive in predicting an adequate response to the TRH stimulation test.

Age and gender distribution of the patients were similar in patients with panhypopituitarism and the control group (Table 1). Free T3, TSH, PRL and PRL responses to TRH stimulation were lower in patients with panhypopituitarism compared to the control group.

**Table 1:** Demographic variables, hormone levels, depressive scores and sexual functions of the study group

	Patient (*n* = 48)	Control (*n* = 20)	*p* value
Age (years)	50 (19–73)	36.5(19–67)	0.157
Gender n (%)			
*Female / Male*	25 (52.1) / 23 (47.9)	9 (45) / 11 (55)	0.395
BMI (kg/m^2^)	27.3 (18.2–46.8)	27.1 (21.6–41.7)	0.586
TSH (µIU/mL)	0.01 (0.0-2.13)	1.19 (0.2–4.51)	**< 0.001**
T4 (ng/dL)	1.30 (0.82–1.83)	1.24 (0.89–1.62)	0.294
T3 (pg/mL)	2.70 (1.73–4.71)	3.27 (2.31–4.19)	**< 0.001**
IGF1 (ng/mL)	41.0 (7.0-247.0)	124.0 (50.9–444.0)	**< 0.001**
Prolactin (ng/mL)	3.36 (0.0–26.0)	10.1 (6.0–23.0)	**< 0.001**
TRH-PRL(*0th minute*) (ng/mL)	3.24 (0.00-29.9)	10.1 (6.0–23.0)	**< 0.001**
TRH-PRL (*20th minute*) (ng/mL)	4.82 (0.16–44.2)	44.8 (18.3–178.0)	**< 0.001**
TRH-PRL (*60th minute*) (ng/mL)	4.28 (0.15–29.6)	23.5 (14.3–97.8)	**< 0.001**
Peak Prolactin (ng/mL)	4.82 (0.16–44.2)	44.8 (18.3–178.0)	**< 0.001**
HDL (mg/dL)	43.0 (27.0–99.0)	39.0(33.0–75.0)	0.188
LDL (mg/dL)	109.0 (55.0-252.0)	119.0(31.0-163.0)	0.144
TG (mg/dL)	163.0 (56.0-566.0)	126.0(59.0-232.0)	0.130
Total Cholesterol (mg/dL)	193.0 (105.0-368.0)	176.0 (86.0-246.0)	0.094
Depression	10.0 (2–31)	4 (0–19)	**0.002**
Sexual Function Score	25 females. 23 male	9 females. 11 males	
*Female* *Male*	23.0 (2–92) 17.5 (5–23)	38(2–83) 22 (13–25)	0.432 **0.007**

Data are reported as median (min-max) or n (%). Bolded p indicates a statistically significant difference between groups (*p* < 0.05). BMI: Body mass index; FSH: Follicle Stimulating Hormone; IGF1: Insulin-like Growth Factor 1; LH: Luteinizing hormone; TSH: Thyroid Stimulating Hormone; TRH-PRL: Prolactin Response to Thyrotropin Releasing Hormone

Depression scores were significantly higher in patients with panhypopituitarism. When patients were divided into 2 groups according to PRL deficiency, PRL deficient patients with panhypopituitarism had higher depression scores compared to the control group, but PRL sufficient patients had similar depression scores compared to the control group. The degree of depressive symptoms was similar in the groups (data not shown).

Sexual function scores were found to be significantly lower in male patients with panhypopituitarism, but there was no difference in female patients. When patients were divided into 2 groups according to PRL deficiency, PRL-deficient male patients with panhypopituitarism had lower sexual function scores compared to the control group, but PRL sufficient male patients had similar sexual function scores compared to the control group. The degree of impairment in sexual function scoring were similar in the groups (data not shown). No differences were detected in sexual function scores in female patients.

Demographic variables and hormone levels of the patients with hypopituitarism with and without PRL deficiency are presented in Table 2. Hormone levels were similar in PRL sufficient and insufficient patients except basal and stimulated PRL levels. Table 3 shows the hormone levels of the patients at the time of diagnosis in patients with available data.

**Table 2:** Demographic variables, hormone levels, scores of depression and sexual functions of the study subgroups according to PRL deficiency compared to the control group

	Group 1 Patients with Insufficient PRL (*n* = 42)	Group 2 Patients with Sufficient PRL (*n* = 6)	Group 3 Control (*n* = 20)	p1 value	p2 value	p3 value
Age (years)	52.5 (19–73)	48.0 (21–64)	36.5(19–67)	0.15		
Gender *n* (%)						
*Female/Male*	25 (60) / 17 (40)	0 (0) / 6 (67)	9 (45) / 11 (55)	0.02	0.165	0.866
BMI (kg/m^2^)	27.3 (18.2–46.8)	25.75 (22.2–40.8)	27.1 (21.6–41.7)	0.74		
TSH (µIU/mL)	0.01 (0.0-2.13)	0.08 (0.0-0.42)	1.19 (0.2–4.51)	0.375	**0.014**	**< 0.001**
T4 (ng/dL)	1.32 (0.82–1.83)	1.21 (0.94–1.40)	1.24 (0.89–1.62)	0.285		
T3 (pg/mL)	2.70 (1.73–4.71)	2.62 (1.73–3.40)	3.27 (2.31–4.19)	0.675	**0.031**	**0.001**
FSH (mIU/mL)	0.77 (0.0-10.9)	1.04 (0.0-2.13)	6.42 (1.57–105.0)	0.557	**0.003**	**< 0.001**
LH (mIU/mL)	0.32 (0.0-6.87)	0.28 (0.0-2.07)	8.97 (2.76–52.2)	0.782	**< 0.001**	**< 0.001**
IGF1 (ng/mL)	39.0 (7.0-247.0)	67.0 (15.4–90.0)	124.0 (50.9–444.0)	0.861	0.09	**< 0.001**
Prolactin (ng/mL)	2.46 (0.0–26.0)	16.3 (7.8–26.0)	10.1 (6.0–23.0)	**< 0.001**	0.340	**< 0.001**
TRH-PRL (ng/mL) (***0th minute***)	2.40 (0.0-29.9)	16.9 (8.80–21.0)	10.1 (6.0–23.0)	**< 0.001**	1.000	**< 0.001**
TRH-PRL (ng/mL) (*20th minute*)	3.59 (0.16–31.8)	25.8 (23.1–44.2)	44.8 (18.3–178.0)	**0.009**	0.771	**< 0.001**
TRH-PRL (ng/mL) (*60th minute*)	3.26 (0.15–29.6)	21.5 (16.5–28.6)	23.5 (14.3–97.8)	**0.002**	1.000	**< 0.001**
Peak PRL (ng/mL)	3.59 (0.16–31.8)	25.8 (23.1–44.2)	44.8 (18.3–178.0)	**0.009**	0.771	**< 0.001**
AUC (PRL response)	203.6(8.7–1845.0)	1185.0(976.0-2107.0)	1966.5(920.6–7491.0)	**0.020**	0.686	**< 0.001**
Glucose (mg/dL)	83.5(64–220)	90.5(79–100)	88.5(69–173)	0.176		
HDL- (mg/dL)	43.0(27.0–99.0)	42.0(28.0–62.0)	39.0(33.0–75.0)	0.319		
LDL (mg/dL)	105.0(65.0-252.0)	154.0(55.0-197.0)	119.0(31.0-163.0)	0.097		
TG (mg/dL)	161.0(56.0-566.0)	167.0(109.0-244.0)	126.0(59.0-232.0)	0.319		
Cholesterol (mg/dL)	192.0(126.0-368.0)	226.0(105.0-287.0)	176.0(86.0-246.0)	0.197		
Depression score	11.0 (2–31)	7 (6–11)	4 (0–19)	0.962	11.0 (2–31)	7 (6–11)
Sexual function score						
*Female n = 22/3/9*	23 (2–92)		38(2–83)		0.695	
*Male n = 17 /6/ 11*	18 (5–23)	17 (5–23)	22 (13–25)	0.818	0.137	**0.033**

Data are reported as median (min-max) or n (%). Bolded p indicates a statistically significant difference between groups (*p* < 0.05), p1: difference between groups 1 and 2, p2: difference between group 2 and 3 p3: difference between groups 1 and 3, AUC (PRL): Area Under Curve of Prolactin response to TRH, BMI: Body mass index; FSH: Follicle Stimulating Hormone; IGF1: Insulin-like Growth Factor 1; LH: Luteinizing Hormone; TSH: Thyroid Stimulating Hormone; TRH-PRL: Prolactin Response to TRH

**Table 3:** Hormone levels of the patients at the time of diagnosis compared to the control group

At the time of diagnosis	Patient (*n* = 23)	Control (*n* = 20)	*p* value
Age (years)	53.0 (19–73)	36.5(19–67)	0.161
Gender *n* (%)			
*Female* *Male*	11 (47.8) 12 (52.2)	9 (45) 11 (55)	0.548
TSH (µIU/mL)	1.04 (0.0-7.14)	1.19 (0.2–4.51)	0.443
T4 (ng/dL)	0.58 (0.24–1.08)	1.24 (0.89–1.62)	**< 0.001**
T3 (pg/mL)	1.97 (1.00-3.76)	3.27 (2.31–4.19)	**< 0.001**
FSH (mIU/mL)	2.25 (0.0-7.70)	6.42 (1.57–105.0)	**< 0.001**
LH (mIU/mL)	0.89 (0.0-3.45)	8.97 (2.76–52.2)	**< 0.001**
Estradiol (pg/ml) (*n* = 11)	0.0 (0.0–30.0)	21.9 (0.50–457.0)	**0.022**
Testosteron (ng/dl) (*n* = 12)	4.5 (0.0-323.0)	445.0 (322.0-775.0)	**< 0.001**
Cortisol (µg/dL)	4.42 (0.32-17.0)	11.5 (4.98–18.8)	**< 0.001**
IGF1 (ng/mL)	36.9 (12.6–92.0)	124.0 (50.9–444.0)	**< 0.001**
PRL (ng/mL)	3.75 (0.85-26.0)	10.1 (6.0–23.0)	**0.002**

Data are reported as median (min-max) or n (%). Bolded p indicates a statistically significant difference between groups (*p* < 0.05). ACTH: Adrenocorticotropic hormone; FSH: *Follicle Stimulating Hormone;* IGF1: Insulin-like Growth Factor 1; *LH*: Luteinizing Hormone; TSH: Thyroid Stimulating Hormone

**Fig. 3:**
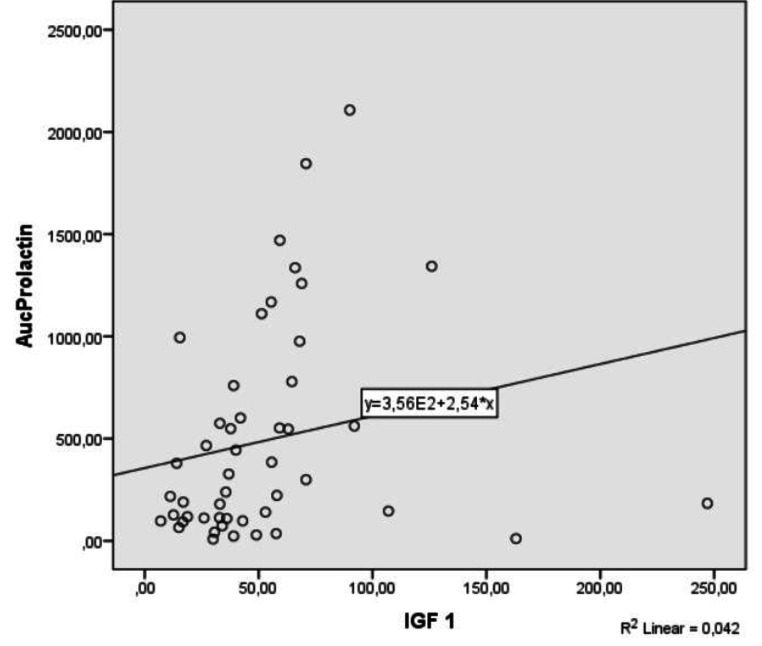
Correlation of IGF-1 levels with AUC (PRL response) to TRH stimulation test (p:0.003, r:0.42)

In the correlation analysis, basal and peak PRL responses to TRH were found to be correlated with IGF-1 levels (p:0.004, r:0.40 and p:0.011, r: 0.36 respectively). Correlation of IGF-1 with AUC (PRL) response to TRH is shown in Fig. 3 (p:0.003, r:0.42).

## Discussion

In addition to presenting the effects of hypoprolactinemia on depression and sexual functions, the present study has demonstrated cut-off levels for basal and stimulated PRL levels for the diagnosis of hypoprolactinemia in different genders. A peak PRL response to TRH of > 18.3 ng/ml in men and > 41.6 ng/ml in women can be accepted as sufficient responses to the test. Basal PRL levels of ≤ 5.7 ng/ml in males and ≤ 7.1 ng/ml in females could predict inadequate responses to the TRH stimulation test with 100% specificity and 70% sensitivity in males and 100% specificity and 80% sensitivity in females. Basal PRL levels of ≥ 8.5 ng/ml in males and ≥ 15.2 ng/ml in females could predict adequate PRL responses to TRH with 100% specificity and 76% sensitivity in males and 96% specifity and 66% sensitivity in females. So, in majority of the patients basal PRL can accurately define the sufficiency of PRL.

As mentioned above, PRL is a neglected hormone in the diagnosis of hypopituitarism. So, although some cut-off levels for basal PRL and PRL response to TRH are defined, not all of them are properly determined. Basal PRL levels below the lower limit of the assay used or some cut-off levels such as 1.8, 2.35, 3, 4 or 5 ng/ml have been suggested [10–14]. TRH stimulation test can also be used for the diagnosis of PRL deficiency [11, 15, 16]. But some of the studies investigated TRH stimulation test in other disorders such as hyperprolactinemia, other pituitary disorders or sellar mass lesions [17–19] or as a combined test for hypopituitarism [15]. The interpretation of TRH stimulation test can be done by calculating R value which is equal to peak PRL minus basal PRL divided by basal PRL. An R value of < 1 (doubling of basal PRL) was suggested to be as insufficient and > 2 as sufficient [19]. If we had used the R value in the present study as a diagnostic criteria, 4 healthy volunteers, 2 patients with sufficient PRL response to TRH and 3 patients with insufficient response to PRL would be included in the indeterminate group (R value:1–2), 2 patients would have a false positive test result and one false negative test result (Online Resource 1). As can be seen from the table (Online Resource 1), a peak PRL level of 2.5 ng/ml can be in the indeterminate zone, on the other hand, a peak PRL level of 31.8 ng/ml can be diagnosed as an insufficient response according to R value. So incremental responses during dynamic stimulation tests can be affected from basal values and sometimes blunted responses can be seen. It doesn’t seem reasonable to consider every blunted PRL response to the TRH test as hypoprolactinemia without taking into account its absolute value.

In an analysis of patients with Sheehan syndrome, we suggested a basal cut-off level of < 4 ng/ml for hypoprolactinemia and > 7.8 ng/ml for normal PRL levels [11]. However, in contrast to the present study, all patients had a diagnosis of Sheehan syndrome and postpartum lactation could be questioned in each of them. On the other hand, since healthy people were not included in the study, reference values ​​for basal PRL were determined according to the history of postpartum lactation and TRH stimulation test, if available [11]. In the present study, we determined the cut-off levels according to TRH stimulation test results in healthy people. Use of basal PRL instead of TRH stimulation test in the diagnosis of hypoprolactinemia is important considering the limited availability of TRH ampul. This can substantially limit the necessity of TRH stimulation test.

Metoclopramide is another pharmacological agent that can increase serum PRL levels. It has been used for differential diagnosis of hyperprolactinemic disorders such as polycystic ovary syndrome or pituitary adenomas [20–22]. However, although metoclopramide may increase PRL levels, it is not a standardized method to evaluate PRL reserve in patients with hypopituitarism.

The causes of panhypopituitarism in the present study were surgery in 31% and surgery + radiotherapy in 10% and Sheehan syndrome in 29%. All of the patients who received combined surgery and radiotherapy or had Sheehan syndrome were PRL deficient. Sheehan syndrome, and combined surgery and radiotherapy were found to be more common in PRL deficient patients. The prevalence of hypoprolactinemia was 87.5% among patients with panhypopituitarism. The number of subjects with sufficient PRL levels (12.5%) was limited in the present study. It is important to note that the study exclusively focused on patients with panhypopituitarism, excluding individuals with acromegaly, prolactinoma and active Cushing disease. These selective inclusion criteria, hence the low number of patients with normal PRL levels might influence the distribution of hypopituitarism etiologies. The prevalence of hypoprolactinemia in patients with a lower number of deficient pituitary hormones is expected to be lower, certainly. In a retrospective study, hypoprolactinemia was found to be 27% among patients with hypopituitarism (13 mild PRL deficiency: 3–5 ng/ml and 14 severe PRL deficiency < 3 ng/ml) [23]. The frequency of hypoprolactinemia is not known well, since there is no clinical need for the diagnosis hypoprolactinemia in daily practice. One of the most common causes of hypoprolactinemia is Sheehan syndrome [4]. Younger age, etiologies such as pituitary apoplexy, craniopharyngioma, and idiopathic hypogonadotropic hypogonadism were suggested to be associated with PRL deficiency [23].

One interesting finding in the present study was the presence of PRL deficiency in all patients with diabetes insipidus. The etiologies of hypopituitarism in these five patients were surgery followed by radiotherapy in all of them (2 for non-functioning pituitary adenoma, 2 for craniopharyngioma and 1 for Cushing disease). Radiotherapy was probably associated with more profound anterior pituitary destruction hence hypoprolactinemia in these patients.

Acquired severe PRL deficiency was suggested to be a marker for more severe hypopituitarism, particularly GH deficiency [13, 23]. This may probably explain the high prevalence of PRL deficiency in the present study, since all included patients had concomitant GH, TSH, ACTH and gonadotropin deficiency. Furthermore, positive correlations of basal and stimulated PRL levels with IGF-1 support this situation. In severe GH deficiency, PRL deficiency was found to be independently associated with low IGF-1 levels. It was suggested that serum PRL might contribute to IGF-1 release in the absence of GH which may explain lower IGF-1 levels in both GH and PRL-deficient patients [14]. However, the role of PRL in IGF-1 generation in healthy people is not known. In the present study, unlike patients with hypopituitarism, no correlations of basal and stimulated PRL levels with IGF-1 were found in the healthy control group.

The effects of true hypoprolactinemia due to destruction of lactotrophs is not known well. Low-normal PRL levels, distinct from hypopituitarism, were found to be associated with type 2 diabetes, metabolic disease and in children with obesity which is reversed after weight loss [12, 24]. Large population-based studies demonstrated the association of low PRL levels with increased prevalence of type 2 DM particularly in women [25, 26]. Presence of PRL receptor is shown to be important for beta cell expansion during pregnancy [27]. Women with gestational diabetes with accompanying low PRL levels during pregnancy were found to have an increased risk of developing type 2 diabetes in their later life [28].

Kyrsiak et al. has shown that cabergoline induced hypoprolactinemia (< 5 ng/ml) was associated with higher levels of 2-h postchallenge glucose, HbA1c level, triglyceride, hsCRP, and lower HDL-cholesterol than patients with normal PRL levels and healthy individuals and these effects were reversible after dose reduction of cabergoline [6]. On the other hand, bromocriptine treatment, which is a dopamine agonist that lowers PRL levels, improves obesity, metabolic syndrome and cardiovascular parameters [29]. The effects of bromocriptine seems to be paradoxical since it both lowers PRL and improves metabolic parameters. So the negative feedback regulation of the dopamine system by PRL may partially explain metabolic abnormalities seen in hypoprolactinemia [30]. In other words, increased PRL leading to increased dopamine, decreased PRL leading to decreased dopamine. In the present study, neither fasting glucose nor lipid levels showed a difference from the control group or in between subgroups of PRL sufficient and insufficient hypopituitary subjects. However, the effects of other pituitary hormone deficiencies and their replacement therapies can also have some confounding effects on glucose metabolism.

Depression scores were found to be higher and male sexual function scores were found to be lower in patients with panhypopituitarism with accompanying PRL deficiency than in healthy control group. Depression scores were higher in patients with hypoprolactinemia than in the control group, but did not show a significant difference from patients with sufficient PRL. So hypoprolactinemia may be associated with higher depressive scores in patients with hypopituitarism. Kyrsiak et al. has previously shown that dopamine agonist-induced subnormal PRL levels were characterised by reduced well-being in both men and women [31, 32]. GH deficiency may also contribute to the depressive symptoms of the patients. To address the effects of other hormone deficiencies, patients underwent evaluation under replacement therapies. However, GH replacement therapy was commenced on to the patients who were willing to receive GH. Because daily requirement of an injectable therapy, concerns of the patients about the side effects of GH when they are informed or lack of clinical benefits after replacement in some patients resulted in discontinuation or not starting GH therapy. It would be perfect if we could reach the targeted IGF-1 levels in all patients to purify the effects of PRL which may be a limitation of the study. Nevertheless, despite being slightly lower in subjects deficient in PRL compared to those with sufficient PRL, IGF-1 levels were similar in both groups reducing the impact of GH on metabolic or other parameters.

Sexual functions were evaluated using valid questionnaires designed for different genders. In the entire hypopituitarism group, male sexual functions were lower compared to the control group. Specifically, in PRL-deficient subjects, male sexual functions were lower than in the control group but similar to PRL-sufficient subjects. Despite testosterone replacement in all male subjects, variations in testosterone levels during replacement and the presence of GH deficiency might still influence sexual functions. Previous findings have indicated that dopamine agonist-induced subnormal PRL levels were associated with impaired sexual functioning in men [31, 32]. Low PRL levels may indeed be associated with sexual dysfunction, and this association could be partially explained by increased dopaminergic tone and a low central serotonin tone [33]. It is important to note that the relationship between hormones, neurotransmitters, and sexual function is complex, and individual responses can vary. Clinical assessments and further research are usually necessary to better understand the specific mechanisms and implications of low PRL levels on sexual dysfunction.

Dopamine agonist-induced sexual dysfunction can also be seen in females [31]. In the present study, female sexual functions do not appear to be significantly affected by hypopituitarism, but since all female patients had PRL deficiency, the effects of hypoprolactinemia could not be evaluated. However, an interesting observation is made regarding the potential influence of societal attitudes on the sexual lives of unmarried female patients, suggesting that conservative mentalities may have inhibitory effects. This raises a question about the validity of the questionnaire in the female group, possibly indicating that social and cultural factors might play a role in shaping perceptions of female sexual functioning. It is crucial to recognize that assessing sexual function involves not only physiological aspects but also psychological and sociocultural factors. Cultural norms, societal expectations, and personal beliefs can influence an individual’s perception of sexual well-being. The validity of questionnaires in assessing female sexual function may indeed be influenced by such factors. Further research may be necessary for a better understanding of the interplay between hormonal factors, societal attitudes, and female sexual functions.

On the other hand, a family analysis, including 3 women with postpartum agalactia (2 sisters and one niece) with a heterozygous mutation identified in the exon 5 of PRL gene, revealed that PRL deficiency can be related to decreased fertility and early menopause, but normal sexual development in females [34].

Recombinant PRL has been used in mothers with lactation insufficiency with or without PRL deficiency and improved milk secretion and content [35]. Lactation is a complex procedure and lack of PRL secretion is seen only in a minority of the mothers. So, other pharmaceutical galactogogues such as domperidone, metoclopramide, GH, metformin and TRH have been tried in the treatment of lactation insufficiency [36]. Acute opioid administration was also shown to increase PRL levels possibly acting through the dopaminergic system [37, 38]. Studies are warranted for defining the therapeutic effects of recombinant PRL, other than lactation, in PRL deficiency.

In conclusion, PRL deficiency is prevalent among individuals with panhypopituitarism, with the potential to result in elevated depression scores in both sexes and impaired sexual functions in males. Notably, the observed lack of PRL effects on BMI, glucose, and lipid levels may be attributed to the limited number of patients with sufficient PRL in the study. A basal PRL level seems to be sufficient for the diagnosis of hypoprolactinemia in routine clinical practice and TRH stimulation test can be reserved for unequivocal results, if it will change our clinical approach, that may presumably occur in the future.

### Electronic supplementary material

Below is the link to the electronic supplementary material.

Supplementary Material 1

## Data Availability

The data that support the findings of this study are not openly available due to reasons of sensitivity and are available from the corresponding author upon reasonable request.
