# Supplementary material for: The diagnosis and prevalence of hypoprolactinemia in patients with panhypopituitarism and the effects on depression and sexual functions
Source: Pituitary. 2024 May 3;27(3):277–86. doi: 10.1007/s11102-024-01393-0 (PMC11150180; doi:10.1007/s11102-024-01393-0)
Supplement: Supplementary file 1 — Supplementary Material 1 [file 11102_2024_1393_MOESM1_ESM.pdf]

Online Resource 1. Basal and peak PRL levels and R value obtained during TRH stimulation test in participants with discordant results

		Basal PRL (ng/ml)	Peak PRL response to TRH stimulation test (ng/ml)	R value	Diagnosis based on peak PRL	Diagnosis based on R value
Control 1	male	11.1	29	1.6	sufficient	indeterminate
Control 2	male	15.8	43.5	1.7	sufficient	indeterminate
Control 3	male	8.56	18.3	1.1	sufficient	indeterminate
Control 4	female	16	46.2	1.9	sufficient	indeterminate
Patient 1	male	8.8	23.1	1.6	sufficient	indeterminate
Patient 2	male	20.9	44.2	1.1	sufficient	indeterminate
Patient 3	male	18.5	24	0.3	sufficient	insufficient
Patient 4	male	29	31.8	0.1	sufficient	insufficient
Patient 5	male	1.1	2.5	1.3	insufficient	indeterminate
Patient 6	male	5.1	10.9	1.2	insufficient	indeterminate
Patient 7	male	1.7	4.6	1.7	insufficient	indeterminate
Patient 8	female	3.9	15.8	2.9	insufficient	sufficient
